# Validation of the HIV Pre-exposure Prophylaxis Stigma Scale: Performance of Likert and Semantic Differential Scale Versions

**DOI:** 10.1007/s10461-020-02820-6

**Published:** 2020-03-10

**Authors:** Aaron J. Siegler, Sarah Wiatrek, Farah Mouhanna, K. Rivet Amico, Karen Dominguez, Jeb Jones, Rupa R. Patel, Leandro A. Mena, Kenneth H. Mayer

**Affiliations:** 1 Department of Behavioral Sciences and Health Education, Rollins School of Public Health, Emory University, 1518 Clifton Rd NE, Atlanta, GA 30322 USA; 2 Department of Epidemiology, Rollins School of Public Health, Emory University, 1518 Clifton Rd NE, Atlanta, GA 30322 USA; 3 Department of Epidemiology, Milken Institute School of Public Health, The George Washington University, 950 New Hampshire Ave NW, Washington, DC 20052 USA; 4 Department of Health Behavior Health Education, School of Public Health, University of Michigan, Ann Arbor, MI 48109 USA; 5 Division of Infectious Diseases, Washington University in St. Louis, 660 South Euclid Avenue, St. Louis, MO 63110 USA; 6 Division of Infectious Diseases, Department of Internal Medicine, School of Medicine, University of Mississippi Medical Center, 2500 North State St, Jackson, MS 39216 USA; 7 Department of Population Health Science, John D. Bower School of Population Health, University of Mississippi Medical Center, 2500 North State St, Jackson, MS 39216 USA; 8 The Fenway Institute, 1340 Boylston Street, Boston, MA USA; 9 Department of Medicine, Beth Israel Deaconess Medical Center/Harvard Medical School, 330 Brookline Avenue, Boston, MA 02115 USA

**Keywords:** Pre-exposure prophylaxis (PrEP), Social stigma, Primary prevention, HIV, Rating scales

## Abstract

Stigma regarding HIV pre-exposure prophylaxis (PrEP) is commonly implicated as a factor limiting the scale-up of this highly effective HIV prevention modality. To quantify and characterize PrEP stigma, we developed and validated a brief HIV PrEP Stigma Scale (HPSS) among a group of 279 men who have sex with men (MSM). Scale development was informed by a theoretical model to enhance content validity. We assessed two scale versions, Semantic Differential and Likert, randomizing the order in which scales were presented to participants. Both scales demonstrated high internal consistency. The Likert scale had substantially better construct validity and was selected as the preferred option. Scale scores demonstrated construct validity through association with constructs of interest: healthcare distrust, HIV knowledge, perceived proportion of friends/partners on PrEP, perceived community evaluation of PrEP, and perceived effectiveness of PrEP. The scale accounted for 25% of the total variance in reported willingness to be on PrEP, indicating the substantial role PrEP stigma may have on decisions to initiate PrEP. Given increased efforts to roll-out PrEP, having a valid tool to determine the level and types of PrEP stigma in individuals, groups, and communities can help direct implementation plans, identify goals for stigma reduction, and monitor progress over time.

**Electronic supplementary material:**

The online version of this article (10.1007/s10461-020-02820-6) contains supplementary material, which is available to authorized users.

## Resumen

El estigma relacionado con la profilaxis pre-exposición para la prevención del VIH (PrEP, por sus siglas en inglés) ha sido identificado como un factor que limita la implementación a gran escala de esta efectiva modalidad de prevención del VIH. Para poder cuantificar y caracterizar el estigma relacionado al PrEP, desarrollamos y validamos una breve Escala de Estigma al PrEP (HPSS, por sus siglas en inglés) con un grupo de 279 hombres que tienen sexo con hombres. El desarrollo de la escala se basó en un modelo teórico para optimizar la validez de contenido. Evaluamos dos versiones de la escala, Diferencial Semántico y Likert, alternando aleatoriamente el orden en que se presentaron las escalas a los participantes. Ambas escalas demostraron alta consistencia interna. La escala Likert tuvo una mejor validez de constructo y fue seleccionada como la opción preferencial. Los puntajes de la escala demostraron validez de constructo a través de su asociación con las siguientes construcciones de interés: desconfianza en el sistema de salud, conocimiento acerca del VIH, percepción de la proporción de amigos/compañeros que toman PrEP, percepción de la evaluación comunitaria acerca del PrEP, y percepción de la efectividad del PrEP. La escala representó el 25% de la varianza total de la disposición a tomar PrEP, lo que indica el importante rol que el estigma asociado al PrEP puede tener en la decisión de empezar a tomar PrEP. Dado el aumento de los esfuerzos para implementar PrEP, el contar con una herramienta válida para determinar el nivel y los tipos de estigma relacionado al PrEP en individuos, grupos y comunidades puede ayudar a dar dirección a los planes de implementación, a identificar objetivos para la reducción del estigma y a monitorear el progreso a lo largo del tiempo.

## Introduction

The use of oral emtricitabine/tenofovir disoproxil fumarate for HIV pre-exposure prophylaxis (PrEP) for men who have sex with men (MSM) has high clinical trial efficacy, providing over 90% protection when the medication is used [1, 2]. Mathematical modeling indicates the promise of PrEP, with one model finding that half of all new HIV infections over a ten-year period could be averted in a population of MSM in the United States if 60% of persons indicated for PrEP were taking the medication with high adherence [3]. Encouragingly, the population-level impact of PrEP is now visible in some settings. In New South Wales, Australia, there was a population-wide 25% decline in new HIV diagnoses after PrEP was made widely and freely available [4].

In the United States, estimates range from 12,000–200,000 individuals taking PrEP in 2017 [5, 6]. Recent gains in increasing PrEP prescriptions may be leveling off, [5] far short of the 1.1 Mio. persons the Centers for Disease Control and Prevention (CDC) estimates are indicated for PrEP [6]. Progress in scaling up PrEP has not been uniform. One metric, the PrEP-to-need ratio, describes disparities in PrEP scale-up by comparing the number of new PrEP prescriptions relative to the number of new HIV diagnoses; the metric identified women, younger persons, and the Southern US region as having low PrEP-to-need ratios [5, 7]. Structural barriers contribute to these disparities; for instance, counties with higher concentrations of residents living in poverty are less likely to have a PrEP-prescribing clinic [9]. Social factors have also been identified as key components limiting PrEP uptake, including relationships with partners, family, and HIV and PrEP stigma [8, 9].

Stigma has been seminally defined by Goffman as a discrediting attribute that leads to “a whole and usual person” to be considered “tainted (and) discounted [10].”Two recent reviews and one meta-analysis identified the stigmatization of PrEP use (hereafter referred to as *PrEP stigma*) as a barrier to PrEP scale-up [11–13]. Individuals that endorsed beliefs stigmatizing PrEP, such as it being “for people who are promiscuous,” were less likely to be interested in taking PrEP [14]. Numerous qualitative studies have identified stigma as a major barrier to accessing and staying on PrEP [8, 15–19].

To best understand PrEP stigma and the ways in which it impacts PrEP decision-making, standardized and validated measurement tools are needed. Past quantitative measurements of PrEP stigma have predominantly consisted of individual belief statements, with each belief statement then assessed for association with PrEP-related outcomes such as willingness to take PrEP [14, 20, 21]. Such items are diverse in content and wording, as well as response format, with some using semantic differential options (*unpleasant ….. pleasant*) [22] and others using Likert scale options (*strongly disagree, disagree, neutral, agree, strongly agree*). With known differences in participant preferences, time and cognitive burden, and impact on psychometric properties, [23–31] response option types used in items and scales assessing PrEP stigma are also important to consider.

Given that stigma may be a substantial barrier to the expanded use of PrEP, there is a need for a validated stigma scale. Based on a stigma theory [32] and with attention to an optimal response option strategy, we developed and evaluated a brief measure of PrEP Stigma- the HIV PrEP Stigma Scale (HPSS).

## Methods

### Participants

Eligible participants were aged 18 or older, male at birth, HIV negative by self-report, and had anal sex with a man in the last 12 months. To prevent fraudulent completions, IP addresses were used to remove duplicate survey responses. Participants were recruited with online banner advertisements on Facebook, a method that does not produce substantially more biased recruitment for MSM than alternative recruitment methods such as venue-based, time–space sampling [33]. Banner advertisements were clicked by 4,137 persons, 1,186 of whom consented to be screened. After completing the eligibility form, 408 were eligible to participate. Among the 393 individuals that consented to participate, 279 completed the survey and were included in the analysis dataset. Consent, screening, and survey activities were conducted on an electronic, HIPAA-compliant survey platform. The study was approved by the Emory University Institutional Review Board, Protocol Number 00,092,291.

## Measures

### Scale Development

To develop the HPSS, we reviewed literature regarding PrEP stigma and identified key sources for items: a study of stigma as a multidimensional barrier, [34] studies of barriers and facilitators to PrEP acceptability, [35, 36] measures from the Adolescent Medicine HIV Trials Network for HIV/AIDS Interventions (ATN) U19 Scale It Up, [37, 38] and measures from an HIV Prevention Trials Network Study, HPTN082 [39, 40]. We sought to include studies representing a broad range of characteristics; in total, there were 1586 participants in these studies, including 402 men, 1171 women, and 7 transgender men; 824 heterosexual, 140 bisexual, and 154 gay/homosexual persons; 666 Black, 250 White, 184 Latino, and 13 Asian; and 400 participants from international settings. These numbers do not represent the full range of the data because not all variables were reported, and not all studies have initiated or completed data collection. Each study reported age differently, yet there was a wide range of ages, with some studies focused on adolescent participation and others on adult participation.

In assessing HPSS measures across these sources, we identified three predominant themes: shame regarding PrEP use, character judgements of people on PrEP, and perceived social support for taking PrEP. In order to develop a diverse set of items, we adapted existing items to address the domains of a stigma framework, comprising internal, anticipated, and experienced stigma [32]. Within each domain, we sought to have at least one item represent one of three attributes of PrEP stigma we discovered in the literature: shame regarding PrEP use, character judgements of people on PrEP, and perceived social support for taking PrEP (Supplement 1). We limited the total number of items on the scale to a small number to facilitate use of the scale in a broad range of settings such as implementation science, program evaluation, and clinical trials research.

### Variables Hypothesized to Correlate with PrEP

To assess construct validity, we tested a set of a priori hypotheses that the Semantic Differential and Likert HPSS would correlate with each of six constructs. Higher levels of PrEP stigma were anticipated to positively correlate with healthcare distrust and negatively correlate with HIV knowledge, willingness to be on PrEP, proportion of friends/partners on PrEP, community evaluation of PrEP, and perceived PrEP effectiveness. The full text of the survey instrument, including all demographic items and constructs detailed below, are provided in Supplement 2.

*Healthcare distrust* was measured with the Health Care System Distrust scale, a 10-item validated instrument that assesses domains such as perceptions of healthcare system honesty and competence [41].

*HIV knowledge* was measured using an 8-item scale from the SHIPP study, developed to incorporate more current understandings of HIV prevention [42]. Questions included more traditional items such as, “the risk for getting HIV is very low when having oral sex” as well as less traditional knowledge items such as, “Nearly all HIV transmission comes from having lots of boyfriends or hook-ups.”

*Willingness to be on PrEP* was measured with an item from the ATN, “How likely would you be to take PrEP in the future?” with Likert response options ranging from very unlikely to very likely [43].

*Perceived proportion of friends/partners on PrEP* was a measure created for this study, assessed by taking the mean of two slider-scale items with a response range of 0–100%: “What proportion of your (‘friends’ OR ‘current and past sexual partners’) are currently taking PrEP?”.

*Community evaluation of PrEP* was assessed with an item developed for this study: “In general, does your community have a positive attitude toward PrEP?” with response options of “Yes”, “No,” and “Unsure.”

*Perceived PrEP effectiveness* was measured with an item from the American Men’s Internet Survey (AMIS): “How effective is PrEP at preventing HIV infection if a person takes their pills every day?” with response options of > 90%, 75–89%, 50–74%, 35–49%, 20–34%, and < 20% [44].

We classified individuals as being eligible for PrEP based on an abbreviation of CDC eligibility criteria we have used previously [45, 46]: HIV negative status and meeting at least one of criteria (1, 2, or 3): (1) had sex with men (not in a monogamous relationship with an HIV-negative partner) and (a) has been diagnosed with an STI and/or (b) had condomless anal sex in the last 6 months, (2) is in an ongoing sexual relation with an HIV-positive partner, and/or (3) injected drugs and (a) shared injection or drug preparation equipment and/or (b) participated in methadone, buprenorphine, or suboxone treatment program in the last 6 months.

### Statistical Analyses

Descriptive statistics of the study sample are presented, followed by an exploratory factor analysis based on maximum likelihood with Promax rotation to determine whether the Semantic Differential HPSS or the Likert HPSS had latent constructs. A cut-off of < 0.4 was adopted to indicate poor factor loadings. Scree plots of Eigenvalues were used to determine the number of factors in each scale. Cronbach’s alpha was used to assess internal consistency reliability of the overall scale. For analyses of scale performance, only participants completing at least 50% of scale items were included. This approach to missing data resulted in excluding a small number of participants for Likert scale assessments (n = 15, 5%) and a larger number of participants for Semantic Differential scale assessments (n = 79, 28%). A number of sensitivity analyses were conducted to determine the impact of missing data (Supplements 5–7), finding no substantial changes in study conclusions. In Table 3, Pearson’s correlation coefficients (r) were used to assess construct validity of the Likert HPSS. Table 4 displays the mean, standard deviation, and beta estimates from bivariate and multiple linear regression models of the Likert HPSS score predicted by a number of socio-demographic and other PrEP-related characteristics. All analyses were performed in SAS 9.4

## Results

From June 8th 2018 to June 14th 2018, 279 participants were enrolled in the study, and their demographic data are presented in Table 1, stratified by the randomized order in which they received the two scale versions. Participants were from 40 different states across the United States, with median 4 participants per state and range 1 to 21 (Supplement 3). Participants were predominantly white (85%), with fewer identifying as Latino (8%), Black (2%), Asian (2%), or multiracial/other (4%). The majority identified as gay (80%). Participants were 13% aged 18–24, 15% aged 25–34, 18% aged 35–49, and 53% aged 50 and above. Over half had college education or higher (61%), and earned greater than $50,000 annually (57%). A minority of participants (9%) were currently taking PrEP.

**Table 1:** Sociodemographic characteristics and HIV-related behaviors of survey participants by randomized order of scale presentation

Variable	Total n (%)	Order: likert, semantic differential^a^ n (%)	Order: semantic differential, likert^a^ n (%)
Age (years)			
< 25	36 (12.9)	20 (13.7)	16 (12.0)
25–35	43 (15.4)	20 (13.7)	23 (17.3)
35–50	51 (18.3)	27 (18.5)	24 (18.1)
> 50	149 (53.4)	79 (54.1)	70 (52.6)
Race/ethnicity			
Black or African-American	6 (2.3)	3 (2.2)	3 (2.4)
White or Caucasian	219 (84.6)	113 (83.1)	106 (85.5)
Hispanic or Latino/a	20 (7.7)	9 (6.6)	11 (8.9)
Asian	4 (1.5)	4 (2.9)	0 (0)
Other	11 (4.2)	7 (5.2)	4 (3.2)
Sexual Orientation			
Heterosexual or straight	3 (1.1)	1 (0.7)	2 (1.5)
Homosexual or gay	221 (79.8)	121 (84)	100 (75.2)
Bisexual	51 (18.4)	21 (14.6)	30 (22.6)
Other	2 (0.7)	1 (0.7)	1 (0.8)
Education			
High School, GED or less	31 (12.2)	16 (11.9)	15 (12.5)
Some college, Associate’s Degree	68 (26.8)	44 (32.8)	24 (20.0)
College, post graduate or professional school	155 (61.0)	74 (55.2)	81 (67.5)
Income			
$0 to $19,999	40 (16.6)	24 (18.8)	16 (14.2)
$20,000 to $49,999	64 (26.6)	33 (25.8)	31 (27.4)
$50,000 to $74,999	59 (24.5)	26 (20.3)	33 (29.2)
$75,000 or more	78 (32.4)	45 (35.2)	33 (29.2)
Health Insurance			
Public	39 (14.8)	22 (18.0)	17 (15.0)
Private	166 (72.2)	83 (68.0)	83 (73.5)
Uninsured	19 (8.3)	13 (10.7)	6 (5.3)
Other	11 (4.8)	4 (3.3)	7 (6.2)
PrEP use			
Never	246 (88.2)	132 (90.4)	114 (85.7)
Current	25 (9.0)	12 (8.2)	13 (9.8)
Previous	8 (2.9)	2 (1.4)	6 (4.5)
Living with HIV	18 (6.8)	9 (6.5)	9 (7.3)
PrEP eligibility^b^	25 (17.9)	15 (19.5)	10 (15.9)
Ongoing sexual relationship with an HIV-positive partner	15 (10.8)	7 (10.1)	8 (11.4)
Had unprotected anal sex in last 6 months	79 (59.9)	37 (58.7)	42 (60.9)
Diagnosed with STI in last 6 months	9 (3.3)	4 (2.8)	5 (3.9)
Injected drugs in last 6 months	2 (0.7)	1 (0.7)	1 (0.8)

*Note*: There were no significant differences between the two order of presentation groups

^a^All participants were provided both Likert and Semantic Differential scale versions. The order of presentation was randomly assigned

^b^PrEP eligibility was determined based on US Preventive Service Task Force Guidelines

Factor analysis of each scale indicated unidimensionality, based on scree plots of Eigenvalues (Supplement 4). Each scale had a single item with a factor loading below 0.4, indicating poor item fit; these two items were removed from all subsequent analyses (Table 2). All remaining items had factor loadings and item-rest correlations above the commonly used threshold of 0.4 [47]. Cronbach’s alpha values indicated high internal consistency, with Semantic Differential 0.88 and Likert 0.82.

**Table 2:** Factor analysis results of likert and continuous scales measuring PrEP stigma

Likert items, 5-point range from ‘strongly disagree’ (1) to ‘strongly agree’ (5)^a^		Mean	SD	Item-rest correlation	Factor 1 loadings
1	I would feel ashamed to take PrEP pills in front of others	2.24	1.06	0.59	0.65
2	Someone taking PrEP should keep their pills hidden	2.19	1.00	0.48	0.56
3	People experience negative judgment because they take PrEP	2.96	1.01	0.43	0.44
4	I would have sex with someone who is taking PrEP^c^	1.95	0.91	0.52	0.62
5	Someone taking PrEP would be seen by others as slutty	2.52	1.07	0.48	0.50
6	People taking PrEP receive praise for being responsible^R^	2.30	0.83	0.48	0.53
7	My *friends* would be supportive of me taking PrEP^c^	2.08	0.84	0.57	0.67
8^b^	Someone taking PrEP would be treated unfairly by their doctors	2.46	1.01	–	–
9	People experience problems when they tell their sex partner(s) they are taking PrEP	2.67	0.91	0.39	0.42
10	I would feel proud to take PrEP every day^c^	2.40	0.93	0.54	0.62
11	People taking PrEP experience verbal harassment	2.58	0.84	0.54	0.53
12	People on PrEP are taking care of their health^c^	1.67	0.72	0.50	0.59
13	My *family* would be supportive of me taking PrEP^c^	2.51	1.07	0.46	0.51
Cronbach alpha					0.837

Semantic differential word pairs, 7-point range from left anchor (3) to right anchor (-3)^a^ “People taking PrEP are …”		Mean	SD	Item-rest correlation	Factor 1 loadings
1	Lazy – – – – – – – motivated	− 1.29	0.91	0.41	0.49
2	Out of control – – – – – – – in control	− 1.13	1.22	0.69	0.75
3	Unfaithful – – – – – – – faithful	− .24	1.32	0.59	0.67
4	Dishonest – – – – – – – Trustworthy	− 1.06	1.06	0.74	0.80
5	Unsupported – – – – – – – supported	− 1.02	1.08	0.70	0.75
6	Ashamed – – – – – – – proud	− 1.07	1.07	0.68	0.78
7	Unattractive – – – – – – – attractive	− .84	1.12	0.57	0.58
8	Risky – – – – – – – safe	− .81	1.47	0.49	0.55
9	Irresponsible – – – – – – – responsible	− 1.30	1.18	0.61	0.62
10^b^	Modest^c^ – – – – – – – promiscuous	.43	1.20	–	–
11	Immoral – – – – – – – moral	− .97	1.12	0.68	0.71
Cronbach alpha					0.884

^a^*Higher* scale values indicate *higher* levels of stigma

^b^Due to the low factor scores (below 0.4), these items were excluded from the final scales, including factor analysis results and all subsequent analyses

^c^Reverse-coded items

Overall, the sample reported low- to moderate-levels of PrEP stigma (Table 2). Scales were coded such that higher values equate to higher levels of stigma. Most participants completed at least half of Semantic Differential items (200/279, 72%), but a greater proportion completed at least half of Likert items (264/279, 95%). Randomization order did not significantly influence rates of item completion; for full detail regarding missingness by item, see Supplements 5 and 6. The overall mean Semantic Differential score was 2.97 on a 7-point scale, approximately 15% (1-point) below the midpoint of the range. The overall Likert scale mean was 2.59 on a 5-point scale, equating to a value slightly closer to *neutral* (3) than to *disagree* (2), approximately 8% (0.41/5.0 scale points) below the midpoint.

**Fig. 1:**
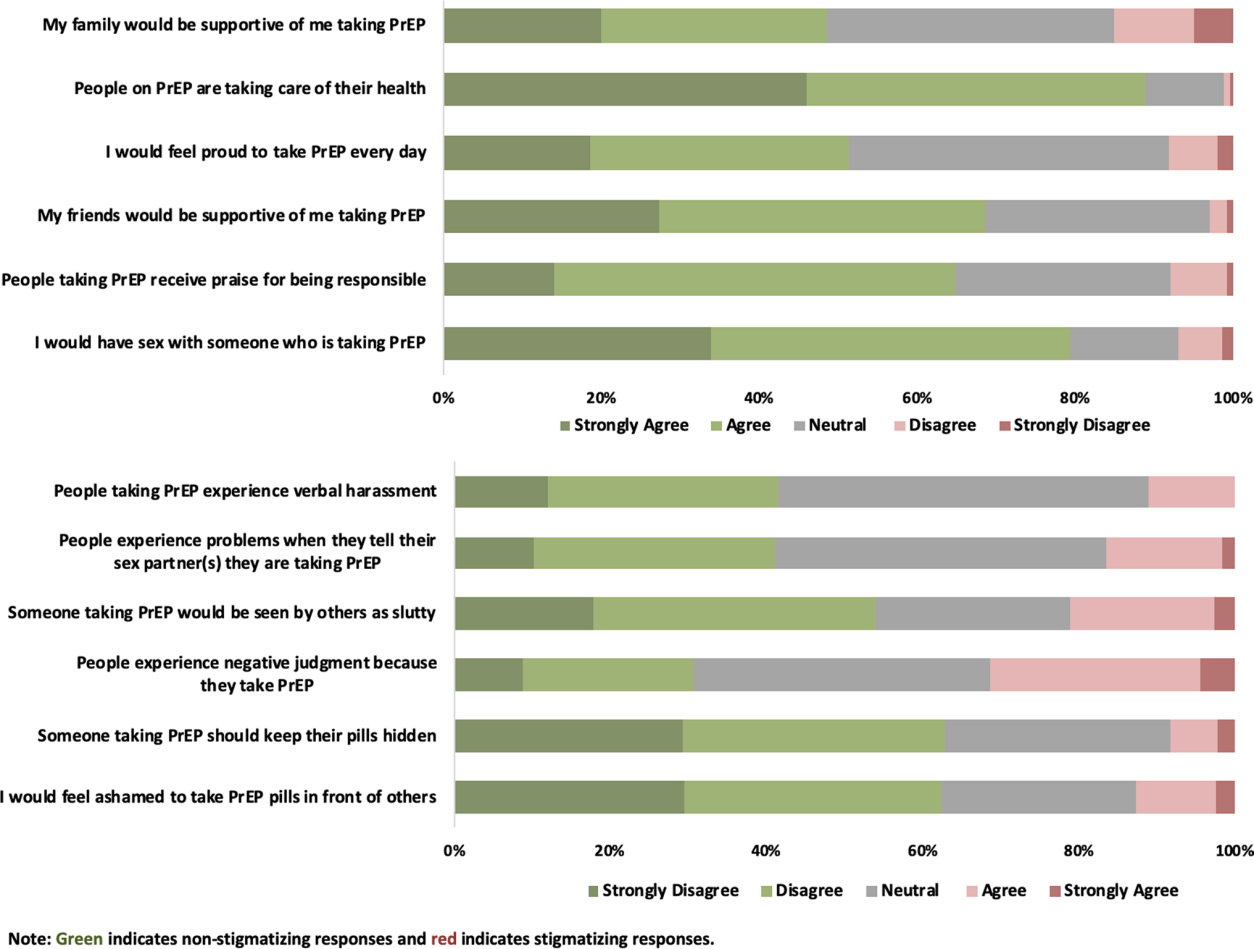
Participant agreement with HIV PrEP Stigma Scale (HPSS) items. *Green indicates non-stigmatizing responses, red indicates stigmatizing responses (Color figure online)

Figure 1 shows the percent distribution of participants’ responses to Likert scale items. Notably, for the majority of items, less than 15% of individuals had responses categorized as stigmatizing. Conversely, many agreed that individuals on PrEP *receive praise* (65%), are *taking care of their health* (89%), and that their *friends would be supportive* (69%). In fact, half would *feel proud to take PrEP*. Yet substantial minorities held stigmatizing views: being *ashamed to take PrEP in front of others* (13%) or anticipating *problems with sex partners* (17%), *being seen by others as slutty* (21%) or *negative judgment* (31%). The mixed views regarding PrEP stigma is perhaps best demonstrated by *neutral* being the most commonly-selected scale category for stigmatizing statements.

The Semantic Differential HPSS was correlated with 2 out of 6 hypothesized variables: willingness to be on PrEP and perceived PrEP effectiveness (Table 3). Both relationships were correlated in the expected direction. The strength of these relationships, based on Cohen’s guidance for interpreting correlation coefficients, was small [48]. The Likert HPSS was correlated with 5 out of 6 hypothesized variables, each in the expected direction. Mistrust in the health care system was correlated with Likert HPSS responses in the expected direction, but was not statistically significant. The strength of the statistically significant correlations with hypothesized variables was small for 1, moderate for 3, and strong for 1. The scale accounted for 25% of the overall variance for the strongly correlated construct, willingness to be on PrEP. A sensitivity analysis in Supplement 7 varied missing data criteria assumptions for Table 3, but identified no substantial differences.

**Table 3:** Likert and Semantic Differential scale correlations with external constructs

Construct	HIV knowledge score	Mistrust in Health care system	Willingness to be on PrEP	Percent of entourage who use PrEP	Community’s positive attitude towards PrEP	Perceived PrEP effectiveness
Likert scale^a,b^						
Pearson’s correlation coefficient	− 0.17	0.11	− 0.51	− 0.36	− 0.42	− 0.29
*p*-value	**0.0046**	0.0702	**< 0.0001**	**< 0.0001**	**< 0.0001**	** < 0.0001**
Semantic Differential scale^a,b^						
Pearson’s correlation coefficient	− 0.09	0.12	− 0.23	− 0.14	− 0.10	− 0.19
*p*-value	0.1856	0.0844	**0.0113**	0.1410	0.2020	**0.0136**

Bold values indicate* p* < .05

^a^*Higher* scale values indicate *higher* levels of stigma

^b^Overall scale mean item scores were calculated and used for assessment of correlations

We assessed whether the Likert HPSS correlated with demographic or other sexual behavior variables and found few associations (Table 4). In unadjusted models, sexual orientation, PrEP use (current or past), and recent STI diagnosis were associated with PrEP stigma. In adjusted models, the same set of variables and income were associated with PrEP stigma. Participants who reported PrEP use had lower PrEP stigma scores by 0.52 and 0.53 points, respectively, compared to those who did not.

**Table 4:** PrEP Stigma Likert scale association with participants’ demographics and HIV-related behaviors

Variable	Mean score^a^	Standard deviation	Unadjusted Beta (95% CI)^b^	Adjusted Beta (95% CI)^b,c^
Age (years)				
< 25	2.33	0.55	REF	REF
25–35	2.34	0.64	0.0 (− 0.25; 0.25)	− 0.12 (− 0.63; 0.40)
35–50	2.35	0.58	0.02 (− 0.22; 0.26)	− 0.17 (− 0.69; 0.35)
> 50	2.34	0.54	0.01 (− 0.20; 0.22)	− 0.14 (− 0.63; 0.35)
Race/ethnicity				
White or Caucasian	2.35	0.54	REF	REF
Black or African-American	2.35	0.95	0.01 (− 0.45; 0.47)	0.02 (− 0.46; 0.49)
Hispanic or Latino/a	2.20	0.58	− 0.15 (− 0.41; 0.12)	− 0.50 (− 1.01; 0.01)
Asian	2.02	0.69	− 0.32 (− 0.89; 0.24)	− 0.80 (− 1.61; 0.02)
Other	2.50	0.67	.16 (− .20; .52)	.23 (− .63; 1.10)
Sexual Orientation				
Homosexual or gay	2.29	0.56	REF*	REF***
Heterosexual or straight	2.84	0.50	0.55 (− 0.08; 1.18)	–
Bisexual	2.53	0.52	0.24 (0.07; 0.41)	0.49 (0.20; 0.78)
Other	2.75	0.71	0.46 (− 0.31; 1.23)	0.58 (− 0.50; 1.65)
Education				
High School, GED or less	2.38	0.58	REF	REF
Some college, Associate’s Degree	2.22	0.55	− 0.16 (− 0.40; 0.09)	0.22 (− 0.21; 0.65)
College, post graduate or professional school	2.38	0.56	− 0.01 (− 0.23; 0.22)	0.56 (0.14; 0.98)
Income				
$0 to $19,999	2.44	0.48	REF	REF^**^
$20,000 to $49,999	2.28	0.54	− 0.16 (− 0.39; 0.07)	− 0.10 (− 0.55; 0.36)
$50,000 to $74,999	2.33	0.54	− 0.11 (− 0.34; 0.13)	0.17 (− 0.29; 0.63)
$75,000 or more	2.29	0.64	− 0.15 (− 0.37; 0.08)	− 0.37 (− 0.85; 0.11)
Health Insurance				
Private	2.33	0.60	REF	REF
Public	2.24	0.44	− 0.08 (− 0.30; 0.13)	− 0.12 (− 0.41; 0.17)
Uninsured	2.53	0.48	0.20 (− 0.07; 0.48)	0.12 (− 0.41; 0.66)
Other	2.34	0.43	.01 (− .35; .37)	.07 (− .45; .59)
Living with HIV				
Yes	2.19	0.49	REF	REF
No	2.35	0.57	0.16 (− 0.11; 0.44)	− 0.04 (− 0.42; 0.35)
PrEP use				
Never	2.41	0.53	REF***	REF***
Current	1.81	0.51	− 0.60 (− 0.82; − 0.38)	− 0.52 (− 0.88; − 0.16)
Previous	1.73	0.46	− 0.68 (− 1.05; − 0.31)	− 0.53 (− 1.05; 0.00)
PrEP eligibility^d^				
Yes	2.43	0.45	REF	–
No	2.42	0.56	− 0.01 (− 0.26; 0.24)	
Ongoing sexual relationship with an HIV-positive partner				
Yes	2.08	0.51	REF	REF
No	2.29	0.57	0.20 (− 0.10; 0.51)	− 0.08 (− 0.48; 0.32)
Had unprotected anal sex in last 6 months				
Yes	2.19	0.63	REF	REF
No	2.38	0.46	0.19 (− 0.01; 0.39)	0.00 (− 0.24; 0.24)
Diagnosed with STI in last 6 months				
Yes	1.74	0.43	REF**	REF*
No	2.37	0.55	0.63 (0.24; 1.02)	0.59 (0.05; 1.12)
Injected drugs in last 6 months				
Yes	2.00	0.35	REF	REF
No	2.35	0.56	0.35 (− 0.44; 1.13)	0.13 (− 1.07; 1.32)

^a^Likert scale score ranges from 1 to 5 with higher score indicating higher stigma

^b^Beta estimates and associated p-values were obtained from bivariate and multivariate linear regression models

^c^Beta estimates were adjusted for all variables in the table except PrEP eligibility which was excluded due to class overlap with eligibility criteria variables

^d^PrEP eligibility was determined based on US Preventive Service Task Force Guidelines

^*^p < .05, **p < .01, *** p < .001

## Discussion

We developed and validated a PrEP stigma scale, the HPSS. In an assessment of Semantic Differential and Likert HPSS versions, both demonstrated face validity by covering key domains in the literature, indicated internal consistency through high Cronbach’s alpha values, and had unidimensionality. The Likert HPSS substantially outperformed the Semantic Differential scale in terms of construct validity, correlating with 5 out of 6 variables hypothesized to be associated with PrEP stigma. It also performed better in terms of completion rates, likely indicating higher acceptability. Of particular relevance to public health programs, the scale accounted for 25% of the variance in participants’ willingness to be on PrEP and 17% of the variance in participants’ perceptions of community attitudes towards PrEP. The strength of these associations indicates that further research regarding PrEP stigma is merited, particularly to better understand the relation between PrEP stigma and willingness to initiate PrEP. This call for further research to develop stigma reduction interventions has been echoed in several recent reviews of PrEP stigma [11–13].

Overall, PrEP stigma was low-moderate in the sample, with the average median HPSS item response slightly closer to *neutral* than to *disagree* for the Likert responses, in which higher values indicated more stigma. A slight majority of participants indicated they would be proud to take PrEP, and strong majorities anticipated support from their friends and families for taking PrEP. Yet a minority of participants anticipated negative consequences, such as being perceived negatively by doctors, experiencing negative judgements, or having problems with sex partners. Despite the relatively moderate levels of overt PrEP stigma, having higher stigma score was strongly associated with lower willingness to take PrEP. Similarly, in a recent study, believing that ‘PrEP is for promiscuous people’ was found to be associated with lower interest in PrEP among black and white MSM in the Southeastern US [14].

The majority of previously published work identified no difference between performance of Semantic Differential and Likert scales, [25–30] yet some studies found Semantic labeling increased performance [23, 24, 30] There are several possible reasons that the Likert scale in our study items outperformed the Semantic Differential scale. First, it was difficult to translate more complex logic of Likert items into the simplified Semantic Differential format we were using. Second, we selected a transformation of items from Likert to Semantic that required greater changes to item wording than some prior studies. These prior studies used nearly identical wording for both scales, changing only the response format. We opted for a more condensed Semantic scale, using a single stem for all items (*People taking PrEP are* …). Higher completion rates for Likert items relative to Semantic Differential items may indicate that such items are more acceptable or interpretable, which conforms to previous findings that participants prefer Likert scale formats [31]. Last, it is possible that the performance across different response formats depends on the specific domains of assessment. Regardless of the reason for the difference, the substantial performance difference identified in this study argues for more use of head-to-head comparisons of scale response formatting.

HPSS was designed based on a stigma framework with three domains (internal, anticipated, and experienced stigma) and on three attributes abstracted from PrEP stigma literature (shame, character judgments, social support), yet our factor analysis revealed a unidimensional scale. The three dimension stigma framework has been validated for HIV stigma for people living with HIV [49, 50] and for substance abuse stigma for people who have substance abuse histories [51]. There are several notable differences between this study and past research. PrEP is a protective behavior, rather than a disease or disorder, potentially influencing scale dimensionality. The HPSS was designed to accommodate both those using and not using PrEP, so responses may be formulated based on various sources: personal experience on PrEP, observed experiences of others on PrEP, discussion among friends/community, online information/discussions, or even supposition. In a sample solely consisting of individuals possessing the stigmatizing trait (PrEP users), responses would likely be primarily based on personal experience and might be multidimensional. Our sample did not include a sufficient number of PrEP users (n = 25) to perform a separate validation with that group. Future work with PrEP users should be conducted to determine if unique subscales emerge and relate to other important outcomes. Regardless of latent constructs, using the stigma framework to structure development of the scale held substantial value by facilitating content validity: ensuring that our items covered diverse topics from different vantages. We found utility for each scale item, with item-rest correlations well above a threshold indicating irrelevance (0.3) and well below a threshold indicating excessive overlap with other items (0.9).

A recently published study among MSM in Chicago developed a PrEP stigma scale and found that Black participants and participants in geographic areas with higher concentrations of HIV incidence had higher levels of PrEP stigma [52]. The scale developed for the Chicago study was published after data collection finished for the present study. There are, however, some key benefits of the scale presented in our study. Most importantly, our study thoroughly assessed construct validity and determined the scale to perform well based on that assessment; the prior scale had no stated assessment of validity. The current study also assessed scale performance across two versions, with findings leading to identification of a scale that substantially outperformed its competitor. It is important to note that there was considerable overlap in item topics across the two scales, including promiscuity, responsibility, and daily use, indicating face validity for each instrument. Future studies may be useful to further assess the relative performance of the two scales.

This study has a number of limitations. First, the sample was majority White and older, and only included MSM; the scale may not perform similarly in other settings. This concern is partially mitigated by the diverse source of studies that provided items that comprise the scale. These studies totaled over 1500 participants, and represented substantial diversity across age, gender, sexual orientation, race, and nationality. We anticipate that the scale will likely perform well across diverse settings, however, future research is needed to provide additional validation across other groups and settings. We are currently investigating how the scale will perform in a study that targets recruitment of over 190 Black MSM in urban areas, [53] and also in a study that targets recruitment of over 120 Latino MSM or Black MSM in rural areas [54]. It is important to note that for the present study, although we did not observe significant differences in mean scale scores by race/ethnicity, we did not have sufficient sample size to adequately assess potential areas of difference. Last, the sample size did not allow for conduct of confirmatory factor analysis: a future assessment should consider confirmatory factor analysis of the scale.

We developed and validated a PrEP stigma scale that is strongly correlated with willingness to be on PrEP. Having a uniform and validated measurement tool, such as the one presented here, can facilitate improved assessment of the impact of PrEP stigma on PrEP initiation and maintenance in care. Moreover, such measurement can help track trends across populations. Goffman notes that, “an attribute that stigmatizes one type of possessor can confirm the usualness of another … stigma then is a relationship between attribute and stereotype.” [10] PrEP is an excellent example of this: PrEP use (attribute) is stigmatized in some communities (negative stereotype), but considered beneficial (positive stereotype) in others [15]. Ongoing assessment of PrEP stigma may help us better understand its influence on PrEP adoption, a potentially vital step in bringing PrEP to scale to dramatically reduce new HIV infections.

## Electronic supplementary material

Below is the link to the electronic supplementary material.

Supplementary file1 (DOCX 123 kb)
