# Supplementary material for: Validation of the HIV Pre-exposure Prophylaxis Stigma Scale: Performance of Likert and Semantic Differential Scale Versions
Source: AIDS Behav. 2020 Mar 10;24(9):2637–49. doi: 10.1007/s10461-020-02820-6 (PMC7423865; doi:10.1007/s10461-020-02820-6)
Supplement: Supplementary file 1 — Supplementary file1 (DOCX 123 kb) [file 10461_2020_2820_MOESM1_ESM.docx]

**Supplement 1. HPSS scale items by stigma attribute and stigma framework domains**

| **Attribute* Stigma Framework**** | | | |
| --- | --- | --- | --- |
|  | **Experienced** | **Anticipated** | **Internalized** |
| Shame regarding PrEP use | People experience negative judgment because they take PrEP.  People taking PrEP experience verbal harassment. | Someone taking PrEP should keep their pills hidden. | I would feel ashamed to take PrEP pills in front of others.  I would feel proud to take PrEP every day.^r^ |
| Character judgments of people on PrEP | People taking PrEP receive praise for being responsible.^r^ | Someone taking PrEP would be seen by others as slutty.^r^ | I would have sex with someone who is taking PrEP.^r^  People on PrEP are taking care of their health.^r^ |
| Perceived social support for taking PrEP | People experience problems when they tell their sex partner(s) they are taking PrEP. | Someone taking PrEP would be treated unfairly by their doctors.*** | My *friends* would be supportive of me taking PrEP.^r^  My *family* would be supportive of me taking PrEP.^r^ |

# r: Reverse-coded item

# *: Stigma attributes identified from a review of PrEP stigma literature

# **: Stigma framework domains from Earnshaw et al.

# ***: Due to low factor scores (below 0.4), this item was excluded from the final scale, including factor analysis results and all subsequent analyses.

**Supplement 2. HPSS Stigma Scale Survey**

Eligibility 2

Demographics 3

PrEP Eligibility 7

Exposure to PrEP 9

PrEP 11

HIV Knowledge 12

PrEP Stigma Likert Scale 13

PrEP Stigma Sematic Differential Scale 16

Health Care Trust/Mistrust 17

PrEP Knowledge 19

## Eligibility Screener

### What is your age?*

_________________________________________________

#### In the past 12 months, have you had sex with a man?*

( ) Yes

( ) No

( ) Don't know

( ) Prefer not to answer

#### What sex were you assigned at birth?*

( ) Male

( ) Female

## Demographics

### Do you identify as Hispanic or Latino/a?

( ) Yes

( ) No

( ) I don't know

#### Which racial group(s) do you consider yourself to be in? Please select all that apply.

[ ] American Indian or Alaska Native

[ ] Asian

[ ] Black or African-American

[ ] Native Hawaiian or Other Pacific Islander

[ ] White

[ ] Other - (please describe): _________________________________________________

#### What is the highest level in school you completed?

( ) College, post graduate or professional school

( ) Some college, Associate’s Degree and/or Technical school

( ) High school or GED

( ) Did not finish high school

#### What best describes your employment status?  Select all that apply.

[ ] Employed full-time

[ ] Employed part-time (Part‐time means that you work less than 35 hours per week during most weeks.)

[ ] Full-time or part-time student

[ ] Unable to work for health reasons

[ ] Not currently employed

[ ] Retired

[ ] Other (please describe):: _________________________________________________

#### What was your income last year from all sources before taxes?

( ) $0 to $9,999

( ) $10,000 to $19,999

( ) $20,000 to $49,999

( ) $50,000 to $74,999

( ) $75,000 or more

#### Do you currently have health insurance? This includes Medicare or Medicaid.

( ) Yes

( ) No

( ) I don't know

#### What kind of health insurance or coverage do you currently have? If you are covered by more than one health insurance plan, please tell us about your PRIMARY plan, the one that you mainly use.

( ) Private health insurance or HMO

( ) Medicaid

( ) Medicare

( ) TRICARE (CHAMPUS)

( ) Veterans Administration Coverage (VA)

( ) I don't know

( ) Other (please describe): _________________________________________________

#### Do you think of yourself as:

( ) Heterosexual or straight

( ) Homosexual or gay

( ) Bisexual

( ) Other (please describe):: _________________________________________________

#### Have you ever been tested for HIV?

( ) Yes

( ) No

( ) I don't know

#### What is your HIV Status?*

( ) HIV-positive

( ) HIV-negative

( ) I don't know/prefer not to answer

## PrEP Eligibility

### The next few questions are a bit personal. Please take your time and answer to the best of your ability.

#### Have you had sex in the past 6 months?

( ) Yes

( ) No

#### In the past 6 months, who have you had sex with?

( ) Men

( ) Women

( ) Both

#### Are you in a monogamous relationship with an HIV-negative partner? A monogamous relationship is when you have no other sex partners and your partner has no other sex partners.

( ) Yes

( ) No

( ) I don't know

#### Do you have an ongoing sexual relationship with an HIV-positive partner?

( ) Yes

( ) No

( ) I don't know

#### In the past 6 months, have you had anal sex without a condom?

( ) Yes

( ) No

#### In the past 6 months, have you been diagnosed with an STI (chlamydia, gonorrhea, syphilis)?

( ) Yes

( ) No

#### In the past 6 months, have you injected drugs that were not prescribed by a clinician?

( ) Yes

( ) No

#### In the past 6 months, have you shared injection or drug preparation equipment (shared needles)?

( ) Yes

( ) No

#### Have you been in a methadone, buprenorphine, or suboxone treatment program in the past 6 months?

( ) Yes

( ) No

#### In the past 6 months, have you exchanged sex for money, drugs or something else like that?

( ) Yes

( ) No

## Exposure to PrEP

### This next section will ask about PrEP.  PrEP is an oral pill taken daily to prevent HIV and requires a doctor’s supervision. Someone would need to test negative for HIV to start taking PrEP and would have come in for refills and check-in’s every three months.

#### Before today, have you ever heard of people taking Pre-exposure prophylaxis (PrEP)?*

( ) Yes

( ) No

( ) I don't know

#### How familiar are you with PrEP?

( ) Very familiar ( ) Somewhat familiar ( ) Minimally familiar ( ) Not at all familiar ( ) I don't know

#### To your best ability, how effective do you think PrEP is at preventing HIV infection if a person takes their pills every day?

( ) More than 90% effective

( ) 75-89% effective

( ) 50-74% effective

( ) 35-49% effective

( ) 20-34% effective

( ) Less than 20% effective

#### What percent of your friends are currently taking PrEP?

0 ________________________[__]_____________________________ 100

#### What percent of your current and past sexual partners are now taking PrEP?

0 ________________________[__]_____________________________ 100

#### In general, does your community have a positive attitude toward PrEP?

( ) Yes

( ) No

( ) I don't know

#### How likely would you be to take PrEP in the future?

( ) Very likely ( ) Somewhat likely ( ) Unlikely ( ) Very unlikely ( ) I don't know ( ) I am currently taking PrEP.

## PrEP

### As a reminder, PrEP is an oral pill taken daily to prevent HIV and requires a doctor’s supervision. Someone would need to test negative for HIV to start taking PrEP and would have come in for refills and check-in’s every three months.

#### Are you currently taking PrEP (e.g. Truvada)?*

( ) Yes

( ) No

#### How long have you been on PrEP? If you have been on PrEP multiple times, just report on your current PrEP regimen/prescription.

( ) Less than 2 months

( ) 2 to 6 months

( ) 7 to 12 months

( ) More than a year

( ) I don't know

### In the last week (7 days), how many days did you miss a dose of PrEP?*

_________________________________________________

#### Have you ever taken PrEP?

( ) Yes

( ) No

( ) I don't know

#### What was the main reason you stopped taking PrEP?

( ) Experienced side effects

( ) No longer at risk for HIV

( ) Access (e.g. cost and services)

( ) Inconvenience to take a pill every day

( ) Stigma associated with taking PrEP

( ) Received a HIV positive test result

#### Would you consider starting PrEP in the next ****six months****?

( ) Yes

( ) No

#### Are you planning to start PrEP in the next ****six months****?

( ) Yes

( ) No

#### Do you plan to begin PrEP in the ****next month****?

( ) Yes

( ) No

#### Have you spoken to a medical provider about starting PrEP?

( ) Yes

( ) No

#### Do you currently have an appointment to talk to a provider about PrEP?

( ) Yes

( ) No

##

## HIV Knowledge

#### Please answer the following statements to the best of your ability even if you have just recently learned about PrEP.

|  | **Yes** | **No** | **I don't know** |
| --- | --- | --- | --- |
| Do you know anyone who has HIV or AIDS? | ( ) | ( ) | ( ) |
| A person who has HIV can look healthy. | ( ) | ( ) | ( ) |
| There is a vaccine that can stop you from getting HIV. | ( ) | ( ) | ( ) |
| The risk for getting HIV is very low for deep kissing (tongue in partner’s mouth) even if your partner has HIV. | ( ) | ( ) | ( ) |

|  | **Yes** | **No** | **I don't know** |
| --- | --- | --- | --- |
| Nearly all HIV transmission comes from having lots of boyfriends or hook-ups. | ( ) | ( ) | ( ) |
| The risk for getting HIV is very low when having oral sex. | ( ) | ( ) | ( ) |
| A person is more likely to get HIV from receptive sex (bottom) than insertive sex (top). | ( ) | ( ) | ( ) |
| Showering or washing your genitals/private parts after having sex will make you less likely to get HIV. | ( ) | ( ) | ( ) |

##

## PrEP Stigma Likert Scale

#### Please answer the following statements to the best of your ability even if you have just recently learned about PrEP. As a reminder, PrEP is an oral pill taken daily to prevent HIV and requires a doctor’s supervision. Someone would need to test negative for HIV to start taking PrEP and would have come in for refills and check-in’s every three months. *****Indicate how much you agree with the following:*****

|  | **Strongly Agree** | **Agree** | **Neutral** | **Disagree** | **Strongly Disagree** |
| --- | --- | --- | --- | --- | --- |
| I would feel ashamed to take PrEP pills in front of others. | ( ) | ( ) | ( ) | ( ) | ( ) |
| Someone taking PrEP should keep their pills hidden. | ( ) | ( ) | ( ) | ( ) | ( ) |
| People experience negative judgment because they take PrEP. | ( ) | ( ) | ( ) | ( ) | ( ) |
| I would have sex with someone who is taking PrEP. | ( ) | ( ) | ( ) | ( ) | ( ) |

|  | **Strongly agree** | **Agree** | **Neutral** | **Disagree** | **Strongly disagree** |
| --- | --- | --- | --- | --- | --- |
| Someone taking PrEP would be seen by others as slutty. | ( ) | ( ) | ( ) | ( ) | ( ) |
| People taking PrEP receive praise for being responsible. | ( ) | ( ) | ( ) | ( ) | ( ) |
| My friends would be supportive of me taking PrEP. | ( ) | ( ) | ( ) | ( ) | ( ) |

|  | **Strongly agree** | **Agree** | **Neutral** | **Disagree** | **Strongly disagree** |
| --- | --- | --- | --- | --- | --- |
| Someone taking PrEP would be treated unfairly by their doctors. | ( ) | ( ) | ( ) | ( ) | ( ) |
| People experience problems when they tell their sex partner(s) they are taking PrEP. | ( ) | ( ) | ( ) | ( ) | ( ) |
| I would feel proud to take PrEP every day. | ( ) | ( ) | ( ) | ( ) | ( ) |

####

|  | **Strongly agree** | **Agree** | **Neutral** | **Disagree** | **Strongly disagree** |
| --- | --- | --- | --- | --- | --- |
| People taking PrEP experience verbal harassment. | ( ) | ( ) | ( ) | ( ) | ( ) |
| People on PrEP are taking care of their health. | ( ) | ( ) | ( ) | ( ) | ( ) |
| My family would be supportive of me taking PrEP. | ( ) | ( ) | ( ) | ( ) | ( ) |

## PrEP Stigma Sematic Differential Scale

#### Using the scale below, select the word that indicates how you feel best describes people who are on PrEP. As a reminder, PrEP is an oral pill taken daily to prevent HIV and requires a doctor’s supervision. Someone would need to test negative for HIV to start taking PrEP and would have come in for refills and check-in’s every three months.

#### Please on PrEP are…

| Lazy ________________________[__]_____________________________ Motivated |
| --- |
| Out of control ________________________[__]________________________ In control |
| Unfaithful ________________________[__]_____________________________Faithful |
| Dishonest ________________________[__]__________________________Trustworthy |
| Unsupported ________________________[__]__________________________Supported |
| Ashamed ________________________[__]_____________________________ Proud |
| Unattractive ________________________[__]___________________________ Attractive |
| Risky ________________________[__]_____________________________Safe |
| Irresponsible ________________________[__]_________________________Responsible |
| Modest ________________________[__]_____________________________Promiscuous |

## Health Care Trust/Mistrust

#### Indicate how much you agree with the following statements:

|  | **Strongly Agree** | **Agree** | **Neutral** | **Disagree** | **Strongly Disagree** |
| --- | --- | --- | --- | --- | --- |
| Medical experiments can be done on me without my knowing about it. | ( ) | ( ) | ( ) | ( ) | ( ) |
| My medical records are kept private. | ( ) | ( ) | ( ) | ( ) | ( ) |
| People die every day because of mistakes by the health care system. | ( ) | ( ) | ( ) | ( ) | ( ) |
| When they take my blood, they do tests they do not tell me about. | ( ) | ( ) | ( ) | ( ) | ( ) |
| If a mistake were made in my health care, the health care system would try to hide it from me. | ( ) | ( ) | ( ) | ( ) | ( ) |

|  | **Strongly agree** | **Agree** | **Neutral** | **Disagree** | **Strongly disagree** |
| --- | --- | --- | --- | --- | --- |
| People can get access to my medical records without my approval. | ( ) | ( ) | ( ) | ( ) | ( ) |
| The health care system cares more about holding costs down than it does about doing what is needed for my health. | ( ) | ( ) | ( ) | ( ) | ( ) |
| I receive high quality medical care from the health care system. | ( ) | ( ) | ( ) | ( ) | ( ) |
| The health care system puts my medical needs above all other considerations when treating my medical problems. | ( ) | ( ) | ( ) | ( ) | ( ) |
| Some medicines have things in them that they do not tell you about. | ( ) | ( ) | ( ) | ( ) | ( ) |

## PrEP Knowledge

### Please read the following scenarios and determine whether you think these *****HIV-negative people***** should start taking PrEP. Marvin is dating Shawn, an HIV positive man. On their first month anniversary, they agree to continue always using condoms when having sex, and to be monogamous. Would PrEP be a good option for Marvin?

( ) Yes

( ) No

#### Ana and Jacob generally have sex after parties where Jacob shoots up (uses intravenous drugs). They have used condoms in the past, but recently they have not used condoms when having sex. Would PrEP be a good option for Ana?

( ) Yes

( ) No

#### Gary often goes out to local bars and clubs to find male sex partners. Most of the time he uses condoms when having anal sex, but he has forgotten a few times. Would PrEP be a good option for Gary?

( ) Yes

( ) No

#### Stanley and Nick have been dating for a two years and have sex without condoms. They decide it is OK to have sex with other men as long as condoms are used every time. Would PrEP be a good option for Stanley?

( ) Yes

( ) No

#### Jane recently learned that John, her husband who she has vaginal sex with, is HIV-positive. They have always used condoms as birth control, and will now use them for HIV prevention too. Would PrEP be a good option for Jane?

( ) Yes

( ) No

#### Tyler went to his doctor for a yearly checkup and was diagnosed with gonorrhea, a sexually transmitted infection. He has not used condoms in the past, but is now planning to always use condoms in the future. Would PrEP be a good option for Tyler?

( ) Yes

( ) No

#### Michael started monogamously dating David 5 years ago. Ever since they both tested HIV negative 4 years ago they have not used condoms for anal sex. Would PrEP be a good option for Michael?

( ) Yes

( ) No

## Thank You!

### Thank you for your interest in our project. Your response is very important to us. Again, we did not collect any identifying information from you (e.g. name, email, phone number) so your responses are confidential.  **Contact Persons** If you have any questions about the study, please contact the investigator in charge, Aaron Siegler at 404-712-9733. If you have questions about your rights or you feel you have been harmed by being in this study, you may contact the Emory Institutional Review Board at (404) 712-0720 or (877)503-9797 or irb@emory.edu.

### **To learn more about PrEP go here:**[https://preplocator.org/](http://preplocator.org)

**Supplement 3. Distribution of study participants by state (N=279)**

| **State name** | **N (%)** |
| --- | --- |
| Mississippi | 1 (0.36) |
| Montana | 1 (0.36) |
| New Mexico | 1 (0.36) |
| North Dakota | 1 (0.36) |
| West Virginia | 1 (0.36) |
| District of Columbia | 2 (0.72) |
| Hawaii | 2 (0.72) |
| Kansas | 2 (0.72) |
| Louisiana | 2 (0.72) |
| Missouri | 2 (0.72) |
| South Dakota | 2 (0.72) |
| Connecticut | 3 (1.08) |
| Nevada | 3 (1.08) |
| Oklahoma | 3 (1.08) |
| Rhode Island | 3 (1.08) |
| South Carolina | 3 (1.08) |
| Tennessee | 3 (1.08) |
| Utah | 3 (1.08) |
| Alabama | 4 (1.43) |
| Arkansas | 4 (1.43) |
| Massachusetts | 4 (1.43) |
| Minnesota | 4 (1.43) |
| New Jersey | 4 (1.43) |
| Oregon | 4 (1.43) |
| Wisconsin | 4 (1.43) |
| Colorado | 5 (1.79) |
| Indiana | 5 (1.79) |
| Kentucky | 5 (1.79) |
| Michigan | 7 (2.51) |
| Washington | 7 (2.51) |
| Iowa | 8 (2.87) |
| Arizona | 9 (3.23) |
| Virginia | 10 (3.58) |
| Illinois | 13 (4.66) |
| Florida | 14 (5.02) |
| Georgia | 14 (5.02) |
| Pennsylvania | 14 (5.02) |
| Texas | 16 (5.73) |
| New York | 17 (6.09) |
| North Carolina | 17 (6.09) |
| California | 20 (7.17) |
| Ohio | 21 (7.53) |
| *Missing State* | 11 (3.94) |
| **TOTAL** | **279 (100)** |

Median=4 participants/state (range=1-21 participants/state)

**Supplement 4a. Scree plot and graph of proportion variance explained by number of factors from factor analysis of Likert scale**


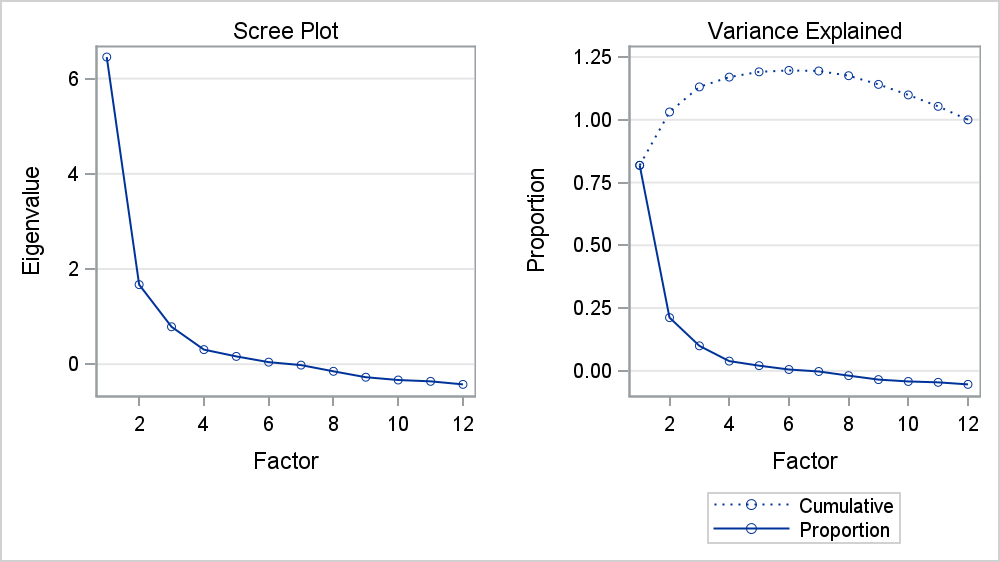


**Supplement 4b. Scree plot and graph of proportion variance explained by number of factors from factor analysis of Semantic Differential scale**


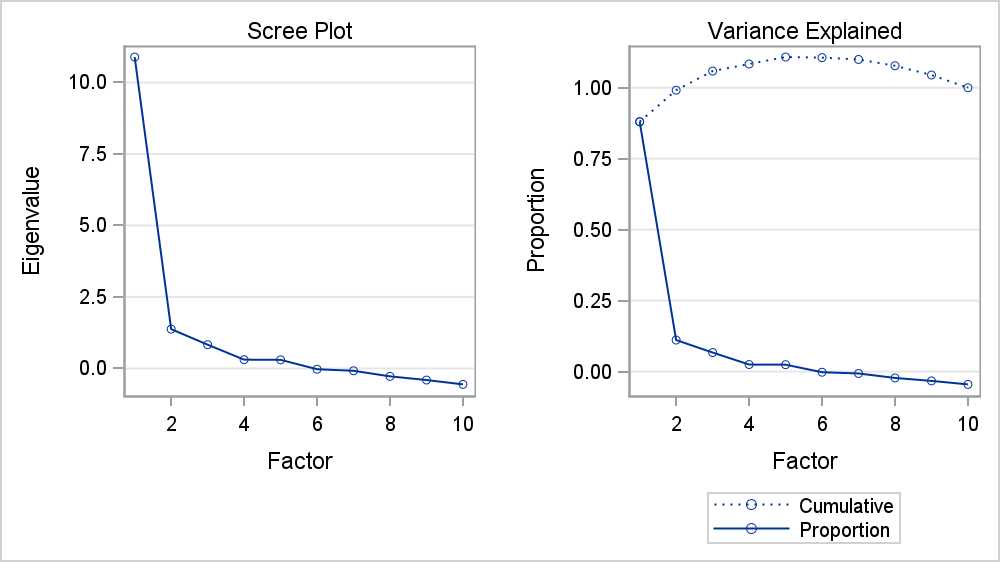


**Supplement 5. Number of missing responses by item for Likert and Semantic scales of PrEP stigma**

|  | N (%) | | |
| --- | --- | --- | --- |
|  | **Total** | **Order: Likert, Semantic Differential^a^** | **Order: Semantic Differential, Likert^a^** |
| ***Likert items, 5-point range from ‘strongly disagree’ to ‘strongly agree’*** | | | |
| I would feel ashamed to take PrEP pills in front of others | 3 (1) | 2 (1) | 1 (1) |
| Someone taking PrEP should keep their pills hidden | 5 (2) | 3 (2) | 2 (2) |
| People experience negative judgment because they take PrEP | 5 (2) | 3 (2) | 2 (2) |
| I would have sex with someone who is taking PrEP^R^ | 7 (3) | 4 (3) | 3 (2) |
| Someone taking PrEP would be seen by others as slutty | 14 (5) | 9 (6) | 5 (4) |
| People taking PrEP receive praise for being responsible^R^ | 15 (5) | 9 (6) | 6 (5) |
| My *friends* would be supportive of me taking PrEP^R^ | 15 (5) | 9 (6) | 6 (5) |
| Someone taking PrEP would be treated unfairly by their doctors | 31 (11) | 16 (11) | 15 (11) |
| People experience problems when they tell their sex partner(s) they are taking PrEP | 31 (11) | 16 (11) | 15 (11) |
| I would feel proud to take PrEP every day^R^ | 32 (11) | 17 (12) | 15 (11) |
| People taking PrEP experience verbal harassment | 35 (13) | 17 (12) | 18 (14) |
| People on PrEP are taking care of their health^R^ | 35 (13) | 17 (12) | 18 (14) |
| My *family* would be supportive of me taking PrEP^R^ | 34 (12) | 16 (11) | 18 (14) |
| ***Semantic Differential word pairs, 7-point range from left anchor to right anchor   “People taking PrEP are …”*** | | | |
| Lazy – – – – – – – Motivated | 57 (20) | 31 (21) | 26 (20) |
| Out of control – – – – – – – In control | 66 (24) | 39 (27) | 27 (20) |
| Unfaithful – – – – – – – Faithful | 88 (32) | 47 (32) | 41 (31) |
| Dishonest – – – – – – – Trustworthy | 85 (30) | 44 (30) | 41 (31) |
| Unsupported – – – – – – – Supported | 92 (33) | 51 (35) | 41 (31) |
| Ashamed – – – – – – – Proud | 92 (33) | 51 (35) | 41 (31) |
| Unattractive – – – – – – – Attractive | 112 (40) | 57 (39) | 55 (41) |
| Risky – – – – – – – Safe | 74 (27) | 40 (27) | 34 (26) |
| Irresponsible – – – – – – – Responsible | 75 (27) | 41 (28) | 34 (26) |
| Modest^R^ – – – – – – – Promiscuous | 105 (38) | 56 (38) | 49 (37) |
| Immoral – – – – – – – Moral | 105 (38) | 54 (37) | 51 (38) |

Note: All participants were provided both Likert and Semantic Differential scale versions. The order of presentation was randomly assigned. Group 1 completed Likert scale first, then Semantic Differential scale and Group 2 the Semantic Differential first, then the Likert scale.

|  |  | Number of participants with following percentages of missing responses by scale: | | | |
| --- | --- | --- | --- | --- | --- |
|  |  | >0% | 50% | 75% | 100% |
| Likert scale | Group 1 | 20 (14) | 9 (6) | 3 (2) | 2 (1) |
|  | Group 2 | 19 (14) | 6 (5) | 2 (2) | 1 (1) |
|  | Total | 39 (14) | 15 (5) | 5 (2) | 3 (1) |
|  | p-value | 0.8882 | 0.5426 | 0.7301 | 0.6139 |
| Semantic scale | Group 1 | 85 (58) | 41 (28) | 32 (22) | 18 (12) |
|  | Group 2 | 80 (60) | 38 (29) | 25 (19) | 9 (7) |
|  | Total | 165 (59) | 79 (28) | 57 (20) | 27 (10) |
|  | p-value | 0.7442 | 0.9281 | 0.5202 | 0.1130 |

**Supplement 6. Number of participants by percentage of missing response to items in the Likert and Semantic scales of PrEP stigma by order of administration of scales in survey**

Note: All participants were provided both Likert and Semantic Differential scale versions. The order of presentation was randomly assigned. Group 1 completed Likert scale first, then Semantic Differential scale and Group 2 the Semantic Differential first, then the Likert scale.

**Supplement 7. Likert and Semantic Differential scale correlations with external constructs by varying exclusion criteria based on percent missing responses to items**

|  |  |  | **HIV knowledge score** | **Mistrust in Health care system** | **Willingness to be on PrEP** | **Percent of entourage who use PrEP** | **Community’s positive attitude towards PrEP** | **Perceived PrEP effectiveness** |
| --- | --- | --- | --- | --- | --- | --- | --- | --- |
| ***Likert scale^a,b^*** | | | | | | | | |
| Excluding participants with: | >0% of items missing | Pearson’s correlation coefficient | -0.16 | 0.13 | -0.52 | -0.37 | -0.42 | -0.30 |
|  |  | *p-value* | **0.0128** | 0.0526 | **<0.0001** | **<0.0001** | **<0.0001** | **<0.0001** |
|  | < =50% of items missing | Pearson’s correlation coefficient | -0.17 | 0.11 | -0.51 | -0.36 | -0.42 | -0.29 |
|  |  | *p-value* | **0.0046** | 0.0702 | **<0.0001** | **<0.0001** | **<0.0001** | **<0.0001** |
|  | < =75% of items missing | Pearson’s correlation coefficient | -0.18 | 0.13 | -0.51 | -0.36 | -0.41 | -0.28 |
|  |  | *p-value* | **0.0038** | **0.0332** | **<0.0001** | **<0.0001** | **<0.0001** | **<0.0001** |
| ***Semantic Differential scale^a,b^*** | | | | | | | | |
| Excluding participants with: | >0% of items missing | Pearson’s correlation coefficient | -0.19 | 0.14 | -0.25 | -0.18 | -0.04 | -0.18 |
|  |  | *p-value* | **0.0412** | 0.1269 | **0.0418** | 0.1832 | 0.7161 | 0.0818 |
|  | < =50% of items missing | Pearson’s correlation coefficient | -0.09 | 0.12 | -0.23 | -0.14 | -0.10 | -0.19 |
|  |  | *p-value* | 0.1856 | 0.0844 | **0.0113** | 0.1410 | 0.2020 | **0.0136** |
|  | < =75% of items missing | Pearson’s correlation coefficient | -0.09 | 0.09 | -0.22 | -0.14 | -0.07 | -0.22 |
|  |  | *p-value* | 0.1882 | 0.2085 | **0.0134** | 0.1500 | 0.3692 | **0.0029** |

^a^ *Higher* scale values indicate *higher* levels of stigma

^b^ Overall scale mean item scores were calculated and used for assessment of correlations.

Note: bolded p-values are statistically significant at α=0.05.
